# Strain-rate sensitive ductility in a low-alloy carbon steel after quenching and partitioning treatment

**DOI:** 10.1038/s41598-019-53303-1

**Published:** 2019-11-19

**Authors:** Philipp Frint, Till Kaiser, Thomas Mehner, Enrico Bruder, Mario Scholze, Bohuslav Mašek, Thomas Lampke, Martin F.-X. Wagner

**Affiliations:** 1 Institute of Materials Science and Engineering, Chemnitz University of Technology, Erfenschlager Str. 73, 09125 Chemnitz, Germany; 2 Physical Metallurgy (PhM), TU Darmstadt, Alarich-Weiss-Str. 2, 64287 Darmstadt, Germany

**Keywords:** Mechanical engineering, Mechanical properties, Metals and alloys

## Abstract

We investigate an extraordinarily high ductility in a low alloy carbon steel at an elevated temperature after a quenching and partitioning (Q&P) treatment. The conventional (quenched and tempered) reference material does not show similar behavior. Interestingly, the Q&P treated material’s ductility is considerably reduced at increasing strain rates while strength remains almost constant. These results indicate the presence of a diffusion-controlled deformation mechanism at elevated temperatures. Our research shows that interlath retained austenite is more stable during deformation at higher temperatures, resulting in a delayed transformation to martensite and therefore to a more pronounced contribution to plastic deformation at (and in the vicinity of) the many interfaces inherently present in this multi-phase steel.

## Introduction

Mankind has used steels for several millennia – yet the search for new steel alloys with tailor-made properties (most notably: high strength, since steels are the most important materials for load-bearing applications) is far from over. Modern materials science contributes substantially to the improvement of steels because it provides a detailed understanding of the complex microstructures that are observed in steels, and that can be substantially modified by thermo-mechanical treatments. The latest generation of so-called advanced high-strength steels exhibit impressive ultimate tensile strengths far exceeding 1 GPa. Even more importantly, the old paradigm that “hardening a steel makes it brittle” has been overcome by the development of multi-phase steels. The quenching and partitioning (Q&P) method addressed in this paper has been specifically developed for tailoring excellent mechanical properties of conventional low-carbon steels by generating microstructures that contain a mixture of martensite and retained austenite^1–7^. Q&P processing typically starts with austenitization, followed by quenching below the martensite start temperature of the investigated steel. The quenching temperature (which is above martensite finish temperature) directly affects the fraction of retained austenite. The subsequent treatment involves carbon partitioning from supersaturated martensite to retained austenite, which results in its (partial) stabilization^8^. Early studies dealing with Q&P steels were focused on thermodynamic aspects like carbon partitioning and on the role of alloying elements like silicon^9–11^ as well as on carbide precipitation^12^. Moreover, multiple microstructural deformation mechanisms have been shown to be involved during plastic deformation of Q&P steels. These include dislocation slip, interface plasticity, and the stress-induced transformation of nanoscale interlath retained austenite^13–16^. A few studies address the effect of strain rate on the deformation behavior of Q&P-processed steels. However, while strain-rate sensitive strength has been repeatedly observed^13,17^, no information is available on the effect of strain rate on the ductility of Q&P steels at ambient and elevated temperatures. Here, for the first time, we investigate an unusually high ductility in a Q&P-processed steel at an elevated temperature, we discuss the effect of strain rate on strength and ductility, and we explain these observations by considering in detail the contributions of different microstructural deformation mechanisms.

## Experimental

A conventionally hot-rolled, low-alloyed carbon steel (thickness 10 mm) was used. The chemical composition was 0.41C, 1.63Si, 1.37Cr, 0.67Mn and Fe bal. (wt%) with martensite start and finish temperatures of 320 °C and 151 °C, respectively. Two different heat-treatment conditions – quenched and tempered (Q&T), and quenched and partitioned (Q&P) – were investigated. Q&T-processing consisted of 3 stages: full austenitization at 950 °C for 21 min, followed by water-quenching to 50 °C and tempering at 250 °C for 35 min (including 25 min for completely heating up the material after placing it in a furnace). For Q&P-processing, 4 stages were applied: full austenitization at 950 °C for 21 min, quenching in ambient temperature water until the material had cooled to 350 °C followed by quenching in a salt bath to 170 °C and holding for 5 min, and finally partitioning at 250 °C for 35 min (including 25 min for heating up). This heat treatment results in a retained austenite fraction of 11 wt%.

To characterize the effects of strain rate and temperature on the deformation behavior, tensile specimens with a gauge length of 20 mm and a diameter of 4 mm were machined parallel to the rolling direction. Tensile tests were performed with both heat-treatment conditions (Q&T, Q&P) at room temperature (293 K) and at an elevated temperature (473 K). Heating up of the tensile specimens was conducted in a conventional radiation furnace (Maytec, Germany) which is built around the testing machine. In order to reach 473 K, a heating duration of 30 min and no additional holding time was used. As the testing temperature is 50 K below the partitioning temperature, only negigible microstructural changes are expected to occur in the heating stage of the experiment prior to tensile testing. Initial strain rates between 10^−4^ and 10^−1^ s^−1^ were applied using a standard Zwick/Roell (Germany) 100 kN tensile testing machine with a tactile extensometer and a standard load cell. Three tensile tests for each combination of material condition, testing temperature and applied strain rate were performed. Additional tensile jump tests were performed using strain rates between 10^−4^ and 10^−3^ s^−1^ to characterize the strain rate sensitivity of the investigated conditions.

A Zeiss NEON scanning electron microscope (SEM) with an acceleration voltage of 5 kV, a working distance of 3.9 mm and a secondary-electron detector was used to analyze typical microstructural features. Additional high-resolution electron-backscatter diffraction (EBSD) data were recorded with a Tescan MIRA3 SEM microscope in a region of 6 × 12 µm² using an acceleration voltage of 15 kV, a beam intensity of 300 nA and a step size of 15 nm. To investigate the austenite distribution, EBSD analyses were conducted using NPAR (Neighbor Pattern Averaging and Reindexing) post-processing to improve the signal-to-noise ratio without additional cleanup procedures.

A complementary quantitative determination of the retained austenite fraction was performed by X-ray diffraction (XRD) utilizing a Siemens D5000 X-ray diffractometer with Co-Kα radiation (35 kV, 30 mA) and point focus. A collimator with 1 mm diameter and a scintillation counter were used. For recording diffraction diagrams, a 2θ-diffraction angle range of 20° to 130° was used with a step size of 0.02° and 15 s/step, resulting in a total measurement time of 23 h for each specimen. Quantitative analyses of retained austenite were performed using the Rietveld method. At least 3 measurements were included in each XRD analysis.

## Results and Discussion

**Figure 1:**
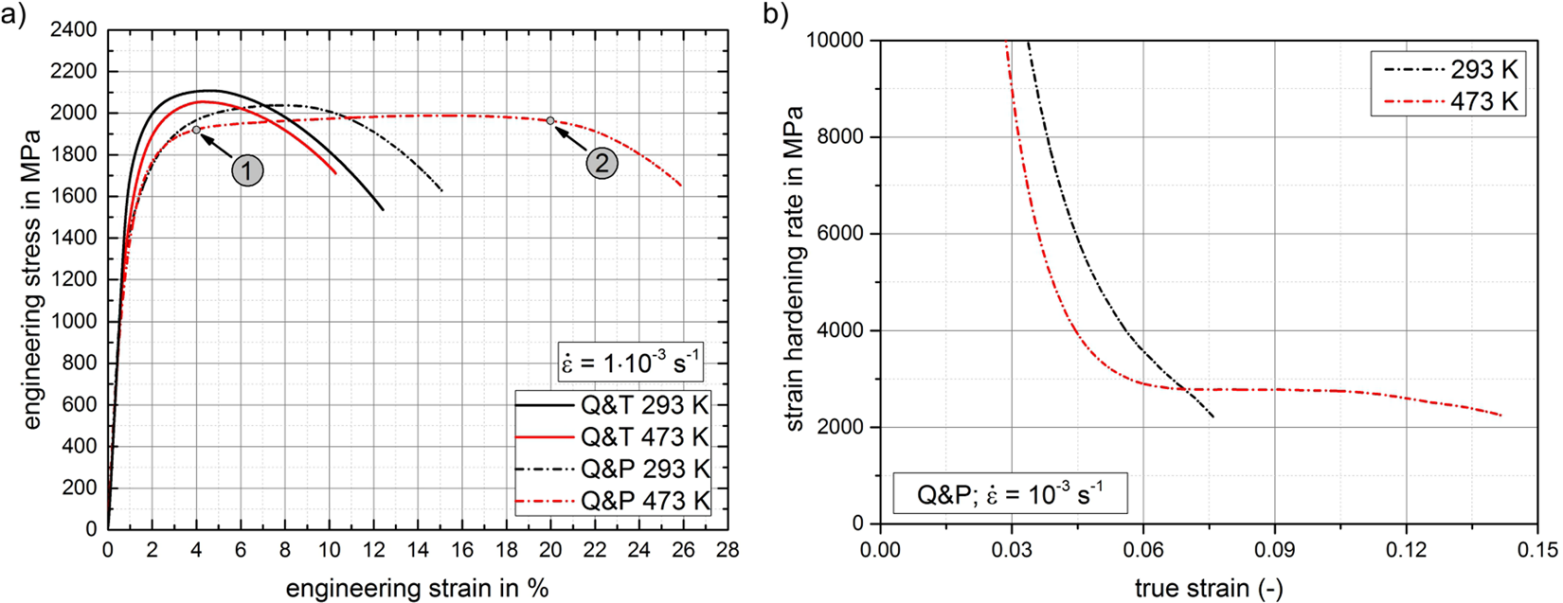
(**a**) Engineering (tensile) stress-strain curves after Q&T and Q&P heat-treatment, at testing temperatures of 293 K and 473 K under quasi-static loading conditions (10^−3^ s^−1^). (**b**) Strain hardening rate vs. true strain highlighting the extraordinary, almost constant strain hardening rate of the Q&P-processed material at 473 K.

The macroscopic deformation behavior is represented by engineering stress-strain curves for both material conditions and testing temperatures under quasi static loading (10^−3^ s^−1^), Fig. 1a. For all investigated conditions, remarkably high strengths in terms of yield strength and ultimate tensile strength were observed (Q&T: 1740/2100 MPa at 293 K, 1520/2050 MPa at 473 K; Q&P: 1390/2050 MPa at 293 K, 1370/2000 MPa at 473 K). For Q&T-processing, there is a slight influence of testing temperature on the yield strength while in case of the Q&P-processed material the yield strength is not affected by the increased temperature.

Comparing the room-temperature results for both conditions, we note a distinct difference of ductility especially in terms of uniform elongation (about 3.5% for Q&T, but 7.0% for Q&P). This result is expected and agrees well with literature, since the larger fraction of retained austenite in the Q&P condition considerably contributes to a Q&P steel’s macroscopic capability to deform plastically. Most importantly, we find a surprisingly large ductility for the Q&P heat treated material at the elevated temperature (473 K). The ductility of the Q&T heat treated material, in contrast, is hardly affected by this temperature increase. The distinct difference in the temperature-dependent deformation behavior of the Q&P condition is especially noticeable in the corresponding strain hardening plots (Fig. 1b). Since the strain hardening behavior in both curves is almost equal up to a deformation of approximately 3% (see also Fig. 1a) and for a better visibility of the interesting region, the plot is limited to hardening rates below 10 GPa. Both curves are plotted until Considère’s instability criterion for the onset of necking (defined as the strain hardening rate becoming equal to the true tensile stress) is met. The hardening rate of the room temperature test shows the typical sharp decline until necking of the specimen occurs. In contrast, at 473 K, the slope of the strain hardening rate considerably changes after a deformation of about 4%. The curve exhibits an extensive plateau characterized by an almost constant strain hardening rate up to the onset of necking at a true strain of 14%. Indeed, engineering stress only drops slowly even after the onset of necking (see Fig. 1a: there is an almost linear region between engineering strains of 4% and 16%, respectively). The existence of this pronounced plateau in the strain hardening curve strongly indicates a change of the deformation mechanisms at elevated temperatures. This result motivated us to further analyze the strain-rate sensitive deformation behavior since temperature-dependent (diffusive) deformation mechanisms also often involve a pronounced strain-rate dependency.

**Figure 2:**
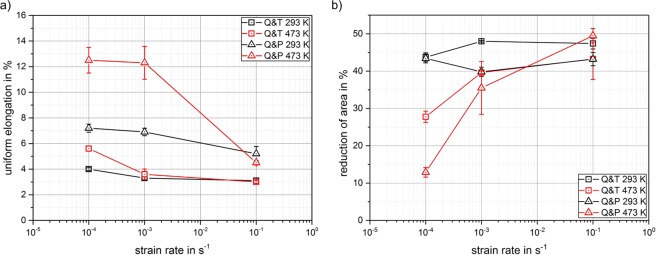
(**a**) Uniform elongation and (**b**) reduction of area (during necking) during tensile testing of both material conditions vs. strain rate at the two testing temperatures (293 K and 473 K).

The strain rate sensitivity parameter $m=\Delta ln\sigma /\Delta ln\dot{\varepsilon }$, which characterizes the sensitivity of a material’s strength to changes of strain rate, was determined from additional jump tests using strain rates between 10^−4^ and 10^−3^ s^−1^. The *m*-values for both material conditions were found to be practically zero at room temperature, indicating a strain rate-independent strength at this temperature. Testing at 473 K also led to low and almost identical average *m*-values for both material conditions (Q&T: 0.0123; Q&P: 0.0118). These results show that the material behavior, when only considered in terms of strength, is hardly strain-rate sensitive. However, our results also indicate a pronounced effect of strain rate on ductility. Figure 2 summarizes the ductility data gained from tensile testing at 3 different strain rates for both temperatures and material conditions. Considering uniform elongation (Fig. 2a) as well as the reduction of the cross-sectional area of necking (Fig. 2b), almost no influence of the applied strain rate at room temperature is found for both material conditions at room temperature. In contrast, a pronounced strain rate sensitivity of ductility is observed for (only) the Q&P-processed material at 473 K: Starting at an average uniform elongation of 12.5% at a strain rate of 10^−4^ s^−1^, this elongation is reduced by more than 50% when testing is performed at 10^−1^ s^−1^. At this strain rate, the uniform elongation coincides with the corresponding value at room temperature. This strain rate-dependent ductility, which to the best of our knowledge has not been observed before in Q&P heat treated steels, strongly indicates that a diffusion-controlled deformation mechanism dominantly acts at elevated temperatures and low strain rates. A similar – but substantially less pronounced – correlation between uniform elongation and strain rate at 473 K also occurs for the Q&T material. In contrast to the decrease of uniform elongation with increasing strain rate, the reduction of area increases (Fig. 2b). This strain-rate sensitive necking behavior is also much more pronounced in the Q&P condition compared to Q&T. These results, again, provide a strong indication that the main deformation mechanisms, especially for the Q&P condition, strongly depend on the temperature and the strain rate.

**Figure 3:**
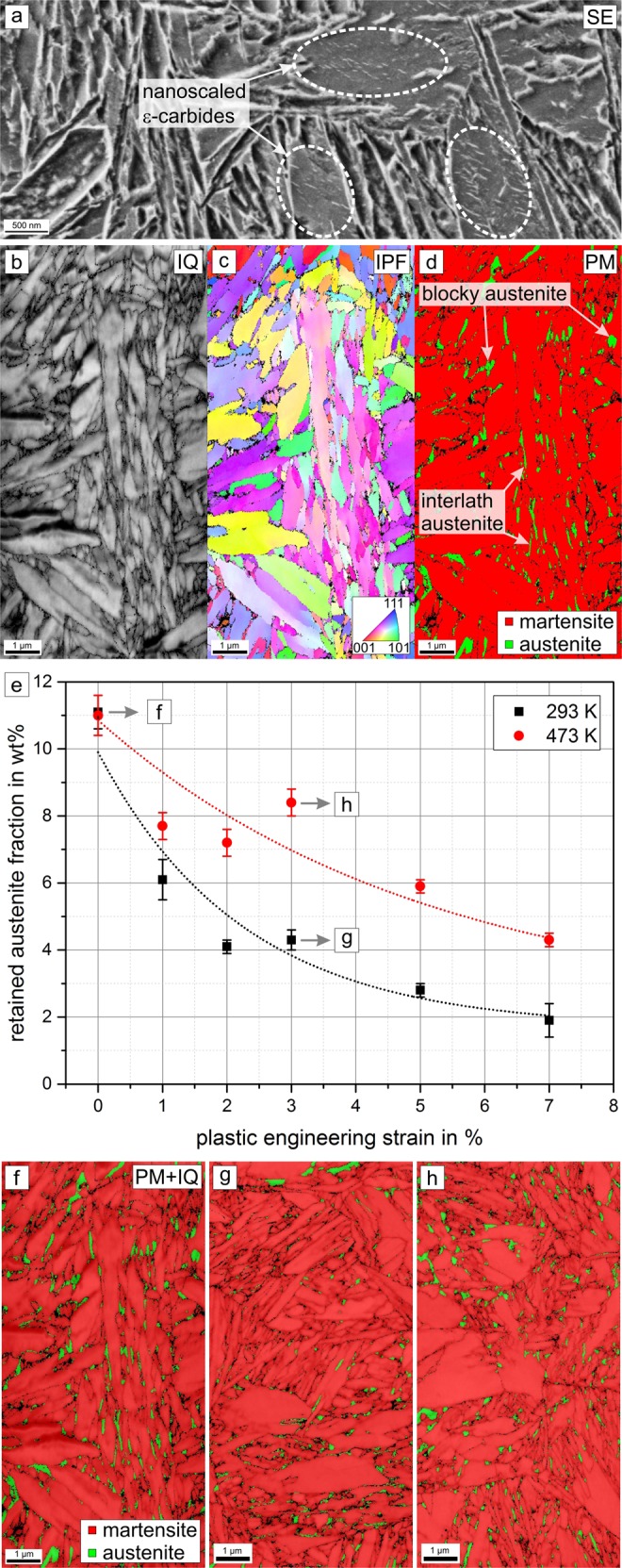
Microstructure of Q&P heat treated steel. (**a**) Secondary electron (SE) contrast reveals the presence of carbides in martensitic areas. Subfigures (**b**–**d**) and (**f**–**h**) show results of ESBD measurements: (**b**) image quality map (IQ), (**c**) inverse pole figure map (IPF) and (**d**) phase map (PM). (**e**) Retained austenite fraction (measured by XRD) vs. (tensile) plastic strain during tensile testing at testing at temperatures 293 K and 473 K. (**f**) PM superimposed with IQ of unstrained microstructure and after 3% tensile plastic strain at g) 293 K and h) 473 K.

It is well documented that the volume fraction and morphology of retained austenite play a key role for the deformation behavior of Q&P heat treated steels^18,19^. To better understand the novel strain-rate sensitive ductility observed here, the microstructure of the Q&P condition was investigated with a focus on the characterization of the retained austenite. The microstructure observed by SEM is generally characterized by typical fine-lath martensite arranged in blocks and packets within the former austenite grain boundaries (see Fig. 3a), in good agreement with^20,21^. Depending on the crystal orientation, in some martensite laths nanoscale precipitates (typical regions highlighted by dashed ellipses in Fig. 3a) can be observed. Most likely these precipitates are ε-carbides^11,12^ that were formed during the comparatively long low-temperature partitioning process. Nanoscale precipitates are effective obstacles for mobile dislocations and therefore precipitation hardening, in addition to the tetragonal distortion of the martensite and grain boundary strengthening, likely contributes to the high strength of the material. To further investigate the distribution of austenite, EBSD analyses were conducted using NPAR post-processing^22^. The image-quality (Fig. 3b) and inverse pole figure maps (Fig. 3c) confirm the small size of the martensite laths shown in the SE image (Fig. 3a). The phase map in Fig. 3d highlights the morphology and distribution of retained austenite (green) in the martensitic matrix (red). A comparison of the phase map and the IPF map reveals that the retained austenite is preferably located at grain boundaries of the martensite laths. Blocky as well as film-like interlath austenite (see Fig. 3d) can be distinguished by considering the aspect ratios, dimensions and locations of the corresponding regions: Blocky retained austenite is typically located at packet boundaries with a size between 100 nm and 500 nm^23^ and a low aspect ratio. In contrast, the film-like interlath austenite is located at the martensite lath boundaries with a size of 10 nm to 50 nm^23^ with typically very high aspect ratios. Interlath austenite plays an important role during plastic deformation in multi-phase steels: It can act as a thin film^14,24^ that can locally accommodate large amounts of plastic deformation by dislocation slip due to its fcc crystal structure. Moreover, interface plasticity driven by sliding of substructure boundaries has been discussed in recent studies^14–16^, and while the corresponding microstructural mechanisms (in particular dislocation-interface interactions) are not fully identified yet, it has been argued^14^ that (film-like) austenite-martensite interfaces between individual martensite laths can also contribute significantly to an accommodation of plastic strains in the context of interface-plasticity. It was also recently shown that retained austenite at triple junctions transforms into martensite at an early deformation stage, whereas interlath retained austenite remains stable up to larger plastic strains^25^. The contribution of interlath retained austenite to strain-rate sensitive ductility, as first observed in the present study, is further discussed in the remainder of this paper.

We note in passing that the absolute fraction of austenite in the microstructure of the Q&P heat treated material cannot be quantified via EBSD data with sufficient accuracy: It is likely that the austenite fraction is underestimated as a consequence of the physical resolution limits, which does not allow the detection of very thin austenite regions in the nanometer range between martensite laths. Therefore, a complementary quantitative determination of the retained austenite fraction was performed by XRD. Using additional specimens from interrupted tensile tests, the retained austenite fractions were determined as a function of plastic strain. For each strain, at least 3 XRD measurements were performed, as summarized in Fig. 3e. The fraction of retained austenite measured via XRD in the initial material is 11 wt% while our EBSD data show a content of 5.6 wt%. This difference obviously results from very small regions of retained austenite (by definition: interlath retained austenite) that cannot be detected by EBSD. An initial fraction of approximately 11 wt% was also measured by XRD in the undeformed part of tensile specimens after testing at both investigated temperatures. This indicates that the thermal influence (heating and subsequent cooling) itself in case of tensile testing at 473 K does not have an impact on the retained austenite fraction, which allows a direct comparison of the fractions after straining at different temperatures. The retained austenite fraction decreases rapidly with increasing plastic strain as a consequence of the transformation to martensite^26^. Both room temperature and elevated temperature curves can be approximated by an exponential decline. The retained austenite fraction during testing at 473 K does not drop as rapidly as it does at room temperature. While, for instance, the austenite fraction at a plastic strain of 3% at room temperature is about 4 wt%, it is still 8 wt% at the same strain at 473 K. Figure 3f–h show representative microstructures (phase maps with superimposed image quality) of the f) unstrained material as well as after 3% tensile deformation at g) 293 K and h) 473 K. These results qualitatively confirm the results of the XRD measurements. Compared to the initial material (Fig. 3f) the fraction of retained austenite is considerably lower after straining at 293 K than at 473 K. At elevated temperatures, the driving force for an austenite-to-martensite transformation is reduced^27^, which results in larger transformation stresses. Thus, the transformation is delayed during tensile straining at 473 K, as confirmed by the increased mechanical stability of austenite^28^ compared to the room temperature experiments. The interlath austenite can hence contribute to deformation up to higher strains, effectively increasing ductility at 473 K. Our results therefore indicate that the increased density and longevity of austenite-martensite interfaces obviously also contributes to more pronounced interface plasticity, as recently discussed, primarily for the case of substructure gliding in lath martensite, in^14,15,24,29^. Since interface plasticity is driven by thermally activated mechanisms near grain/lath boundaries, it predominantly contributes to plastic deformation at elevated temperatures and low strain rates, but less so at lower temperatures and/or higher strain rates, resulting in a considerable reduction of the uniform elongation as shown in Fig. 2a.

**Figure 4:**
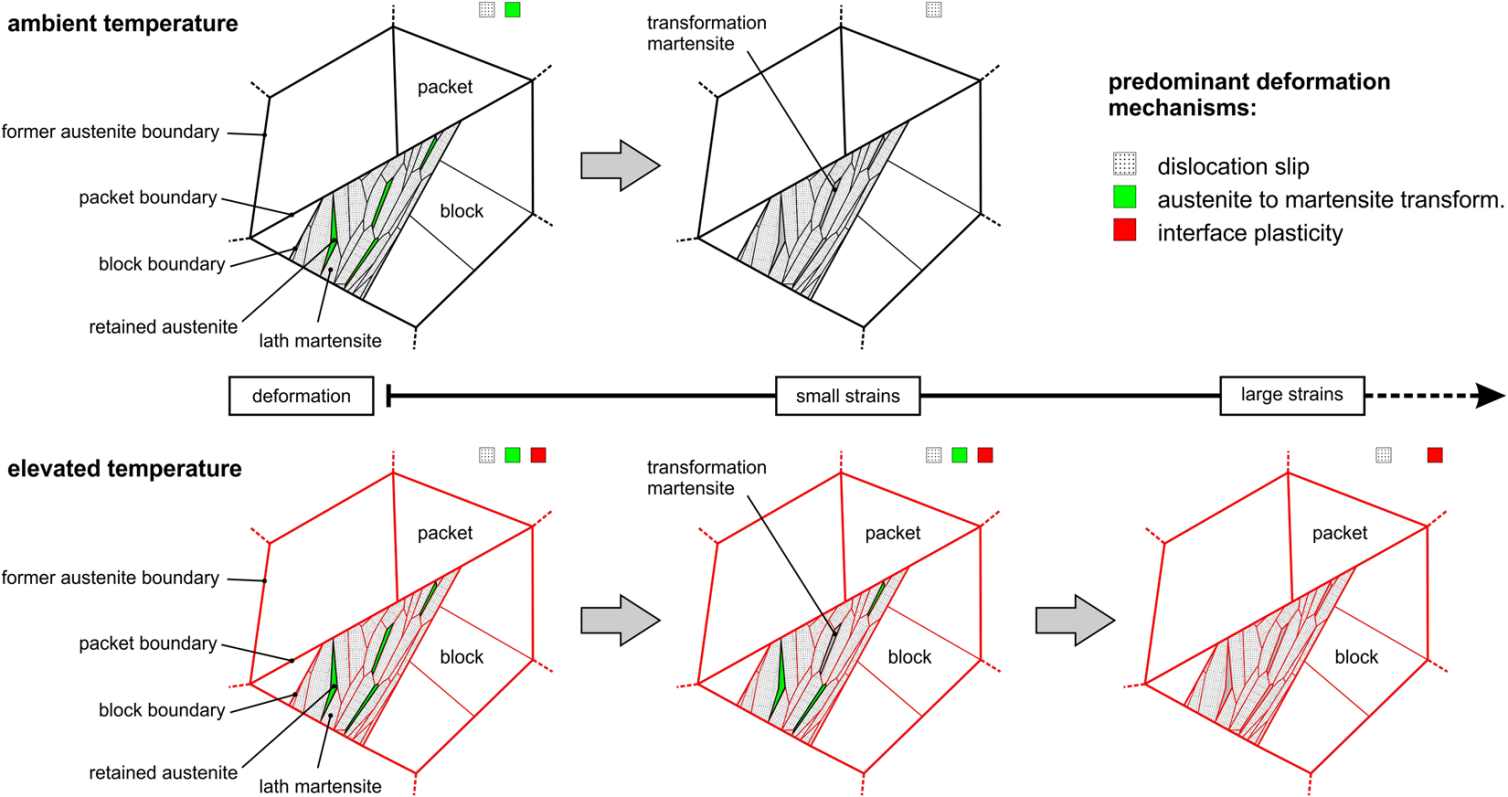
Microstructural model of the key deformation mechanisms that dominate the material behavior at ambient and elevated temperatures. Retained austenite remains stable up to higher macroscopic strains at elevated temperature. Plastic deformation in austenite and the martensitic transformation itself operate up to higher strains. These mechanisms in combination with interface plasticity contribute to the increased ductility at an elevated temperature.

To summarize, we have observed that the ductility of a Q&P heat treated low-alloy carbon steel with excellent mechanical properties exhibits an unusual strain rate sensitivity at elevated temperatures but is almost insensitive to strain rate at room temperature. This novel effect can be attributed to an interplay of several deformation mechanisms. In closing, we discuss a microstructural scenario based on our microstructural observations and XRD measurements in the schematic shown in Fig. 4. For ambient and elevated temperatures, we focus on the relative contributions of (I) dislocation slip and recovery; (II) austenite-to-martensite transformation; (III) interface plasticity, which we define in the broad sense that gliding along interfaces can occur both between martensite laths and austenite-marteniste interfaces. Dislocation slip occurs in both phases and dominates the mechanical behavior at small strains and ambient temperature. At elevated temperature, an equilibrium of strain hardening by dislocation multiplication and dynamic recovery by dislocation annihilation occurs and thus leads to the almost constant strain hardening rate and very high ductility at 473 K. Moreover, diffusion-controlled (and thus strain-rate sensitive) interface plasticity is likely to considerably contribute to the deformation at 473 K, but much less at 293 K. Many different types of interfaces (typically boundaries with high misorientations) are present – most importantly, former austenite grain boundaries, block and packet boundaries. Furthermore, martensite-retained austenite interfaces provide additional sites for interface plasticity and our study highlights that this indeed strongly contributes to the impressive strain-rate sensitive ductility observed here at elevated temperatures. This view is further supported by our experimental observation that interlath retained austenite is more stable during deformation at higher temperatures, which results in a delayed transformation from austenite to martensite and thus promotes both dislocation slip in austenite and interface plasticity up to higher plastic strains, and that these effects are much less pronounced in the Q&T reference condition.
